# Trends in Urogynecology—Transvaginal Mesh Surgery in Germany

**DOI:** 10.3390/jcm13040987

**Published:** 2024-02-08

**Authors:** Yaman Degirmenci, Kathrin Stewen, Anna Dionysopoulou, Lina Judit Schiestl, Konstantin Hofmann, Christine Skala, Annette Hasenburg, Roxana Schwab

**Affiliations:** Department of Gynecology and Obstetrics, University Medical Center of the Johannes Gutenberg University, 55131 Mainz, Germany; kathrin.stewen@unimedizin-mainz.de (K.S.); anna.dionysopoulou@unimedizin-mainz.de (A.D.); lina.schiestl@unimedizin-mainz.de (L.J.S.); konstantin.hofmann@unimedizin-mainz.de (K.H.); christine.skala@unimedizin-mainz.de (C.S.); annette.hasenburg@unimedizin-mainz.de (A.H.); roxana.schwab@unimedizin-mainz.de (R.S.)

**Keywords:** native tissue repair, pelvic organ prolapse, transvaginal mesh surgery, cystocele, rectocele

## Abstract

Background: Pelvic organ prolapse constitutes a prevalent condition associated with a considerable impact on the quality of life. The utilization of transvaginal mesh surgery for managing POP has been a subject of extensive debate. Globally, trends in TVM surgery experienced significant shifts subsequent to warnings issued by the FDA. Methods: This study aims to explore temporal patterns in transvaginal mesh surgery in the German healthcare system. A comprehensive analysis was conducted on in-patient data from the German Federal Statistical Office spanning 2006 to 2021. A total of 1,150,811 operations, each associated with specific codes, were incorporated into the study. Linear regression analysis was employed to delineate discernible trends. Results: The trends in transvaginal mesh surgery within the anterior compartment exhibited relative stability (*p* = 0.147); however, a significant decline was noted in all other compartments (posterior: *p* < 0.001, enterocele surgery: *p* < 0.001). A subtle increasing trend was observed for uterine-preserving transvaginal mesh surgery (*p* = 0.045). Conclusion: Surgical trends over the specified timeframe demonstrate how POP management has evolved globally. Notably, despite observed fluctuations, transvaginal mesh surgery remains a viable option, particularly for specific cases with a high risk of relapse and contraindications to alternative surgical approaches.

## 1. Introduction

Pelvic organ prolapse (POP) is characterized as the descent or displacement of pelvic organs through the vaginal canal [1]. Afflicting up to 50% of women, POP significantly impairs quality of life [2]. While a pelvic organ prolapse can be asymptomatic, it often presents with pelvic floor symptoms such as pressure or dysfunction in the urinary, sexual, and defecatory domains [3]. Therapeutic options for women seeking intervention for this condition span from conservative approaches to surgical procedures [4]. In cases where conservative measures yield unsatisfactory results, various surgical modalities, encompassing both native tissue repair (NTR) and mesh repair, are considered. The lifetime risk of operative repair for pelvic organ prolapse is estimated at approximately 15% [5,6,7], with a cumulative risk of recurrent surgery for a prolapse in another compartment or recurrence at around 30% [5]. Projections indicate a surge of about 25% in the number of women seeking surgical repair for POP from 2020 to 2050 as the population ages [8].

Given the reported high recurrence rates, particularly following traditional vaginal NTR, reaching up to 50%, innovative technologies such as transvaginal mesh repair for POP were developed to improve surgical outcomes [9]. The inaugural product designed explicitly for pelvic organ prolapse repair received approval from the US Food and Drug Administration (FDA) in 2002 [10]. Mesh placement gained popularity in subsequent years as a promising approach to mitigate the risk of recurrence associated with prolapse surgery. However, in response to the escalating trend in transvaginal mesh surgery and the increasing complication rates linked to mesh implants, the FDA issued consecutive warnings in 2008 and 2011 [11,12]. In 2016, the FDA categorized vaginal mesh products as high-risk (class III) and eventually prohibited their sale in 2019 [13].

The prohibition of transvaginal mesh (TVM) implants led to substantial alterations in clinical practices and the management of pelvic organ prolapse (POP) in both the United States and numerous European countries. A dramatic decline in the utilization of transvaginal mesh implants has been observed subsequent to the warnings by the FDA [14]. Over the past two decades, meshes for transvaginal repair of POP have become increasingly controversial. However, in contrast to the FDA, the Scientific Committee on Emerging and Newly Identified Health Risks (SCENIHR), European Urology Association (EAU), European Urogynaecological Association (EUGA), and American Urogynecologic Society have issued positive statements endorsing the use of transvaginal meshes for treating urinary incontinence and pelvic organ prolapse [13]. According to their statements, TVM repair remains a crucial treatment option for POP, and numerous mesh devices designed for POP treatment remain available worldwide.

The objective of our study was to investigate the trajectory of controversial transvaginal mesh surgery within the German healthcare system spanning the years 2006 to 2021.

## 2. Materials and Methods

In this retrospective analysis, we utilized data from the German Federal Statistical Office, which includes information on the annual number of surgeries categorized by surgery codes (OPS codes) for in-patients without specific medical indications. To establish a systematically classified dataset, we examined OPS codes related to transvaginal mesh surgery and vaginal native tissue repair, along with their corresponding codes (Table 1), for the years 2006 to 2021.

The OPS codes undergo annual updates, and the data for anterior and posterior colporrhaphies, with and without mesh repair, were consistently available from 2006 to 2021 with identical OPS codes. Vaginal enterocele surgery data, with and without mesh repair, were definitively and separately recorded in the OPS system starting in 2010, thus being included in the analysis from that year onward. For transvaginal surgery regarding apical prolapse, the OPS system clearly defined the codes starting in 2016, and accordingly, the related data were included in the analysis from 2016.

To evaluate the impact of age distribution on surgery numbers, the results were stratified into six age groups, ranging from under 40 years (<40) to between 40 and 50 years (40–50), between 50 and 60 years (50–60), between 60 and 70 years (60–70), between 70 and 80 years (70–80), and over 80 years (>80). Univariate linear regression analyses with time as the independent factor were performed using SPSS Version 25 to assess the trends of the curves. A *p*-value < 0.05 was considered statistically significant.

According to German laws and regulations, ethics approval was not necessary, as we utilized aggregated and anonymized data.

## 3. Results

A comprehensive analysis was conducted on a dataset encompassing 1,150,811 surgeries, identified by corresponding OPS codes, spanning the period from 2006 to 2021. The predominant surgical interventions were associated with the anterior compartment, primarily anterior colporrhaphy (*n* = 568,911), accounting for 49.3% of the total surgeries. The second most frequent procedures were corrections in the posterior compartment through posterior colporrhaphy (*n* = 453,628), accounting for 39.3% of the surgeries.

The analysis included TVM surgery and NTR within the apical compartment based on uterine fixation, cervical fixation, and vaginal stump fixation (TVM *n* = 38,285; NTR *n* = 59,681). Transvaginal enterocele treatment, represented by a specific OPS code since 2010, was considered for cases between 2010 and 2021 (*n* = 33,306), constituting 3% of the total.

**Figure 1:**
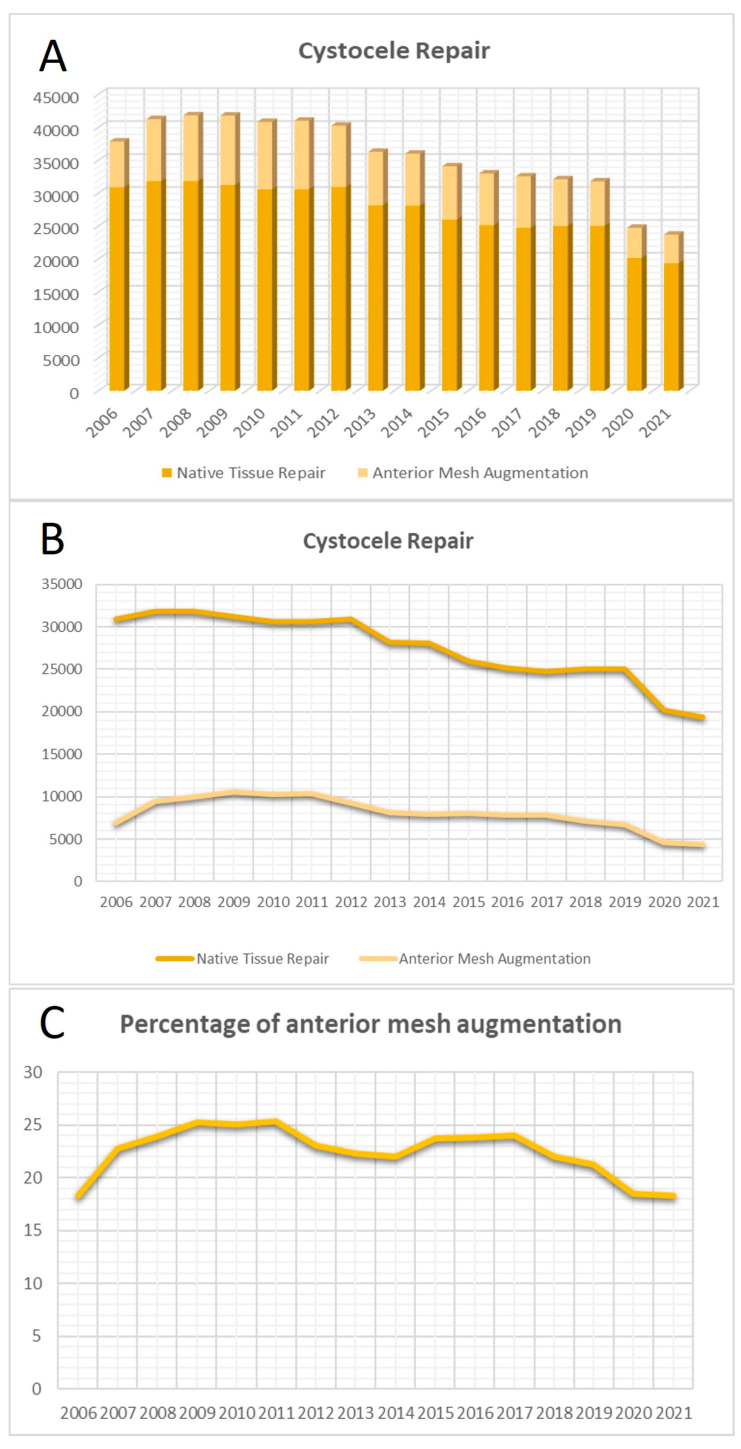
Surgical trends in anterior repair. (**A**) Total number of anterior colporrhaphies over the years. (**B**) Curves of the surgeries with native tissue repair (NTR) and transvaginal mesh (TVM) in the anterior compartment. (**C**) Curve of the proportion of TVM surgeries in the anterior compartment.

In the context of anterior colporrhaphy, there was a noteworthy reduction in the overall number of vaginal operations from 2006 to 2021 (β = −0.901, *p* < 0.001, R^2^ = 0.81). This decline was observed independently for both NTR (β = −0.938, *p* < 0.001, R^2^ = 0.87) and TVM (β = −0.749, *p* < 0.05, R^2^ = 0.56). The percentage of TVM surgeries in the anterior compartment remained stable over the years (β = −0.380, *p* = 0.147; R^2^ = 0.14) (Figure 1).

**Figure 2:**
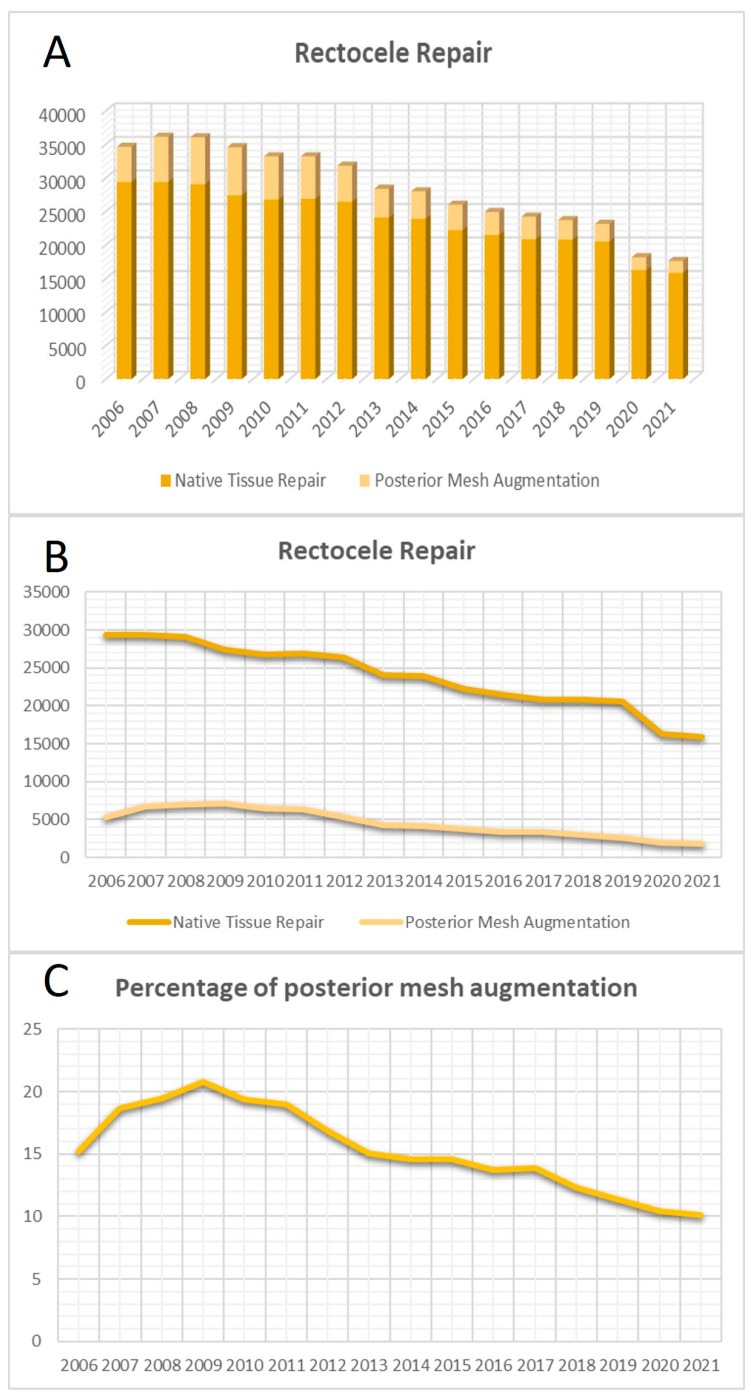
Surgical trends in posterior colporrhaphies. (**A**) Total number of posterior colporrhaphies over the years. (**B**) Curves of the surgeries with native tissue repair (NTR) and transvaginal mesh (TVM) in the posterior compartment. (**C**) Curve of the proportion of TVM surgeries in the posterior compartment.

Similar to vaginal surgeries in the anterior compartment, procedures addressing rectocele within the posterior colporrhaphy context displayed a notable reduction between 2006 and 2021 (β = −0.975, *p* < 0.001, R^2^ = 0.95). Concurrent with the overall decline in the total number of operations in the posterior compartment, both NTR and TVM surgeries in the posterior compartment significantly decreased from 2006 to 2021 (NTR: β = −0.977, *p* < 0.001, R^2^ = 0.95; TVM: β = −0.928, *p* < 0.001, R^2^ = 0.86). Unlike anterior colporrhaphy, the utilization of TVM in the posterior compartment exhibited a significant decrease over the years (β = −0.869, *p* < 0.001, R^2^ = 0.75) (Figure 2).

**Figure 3:**
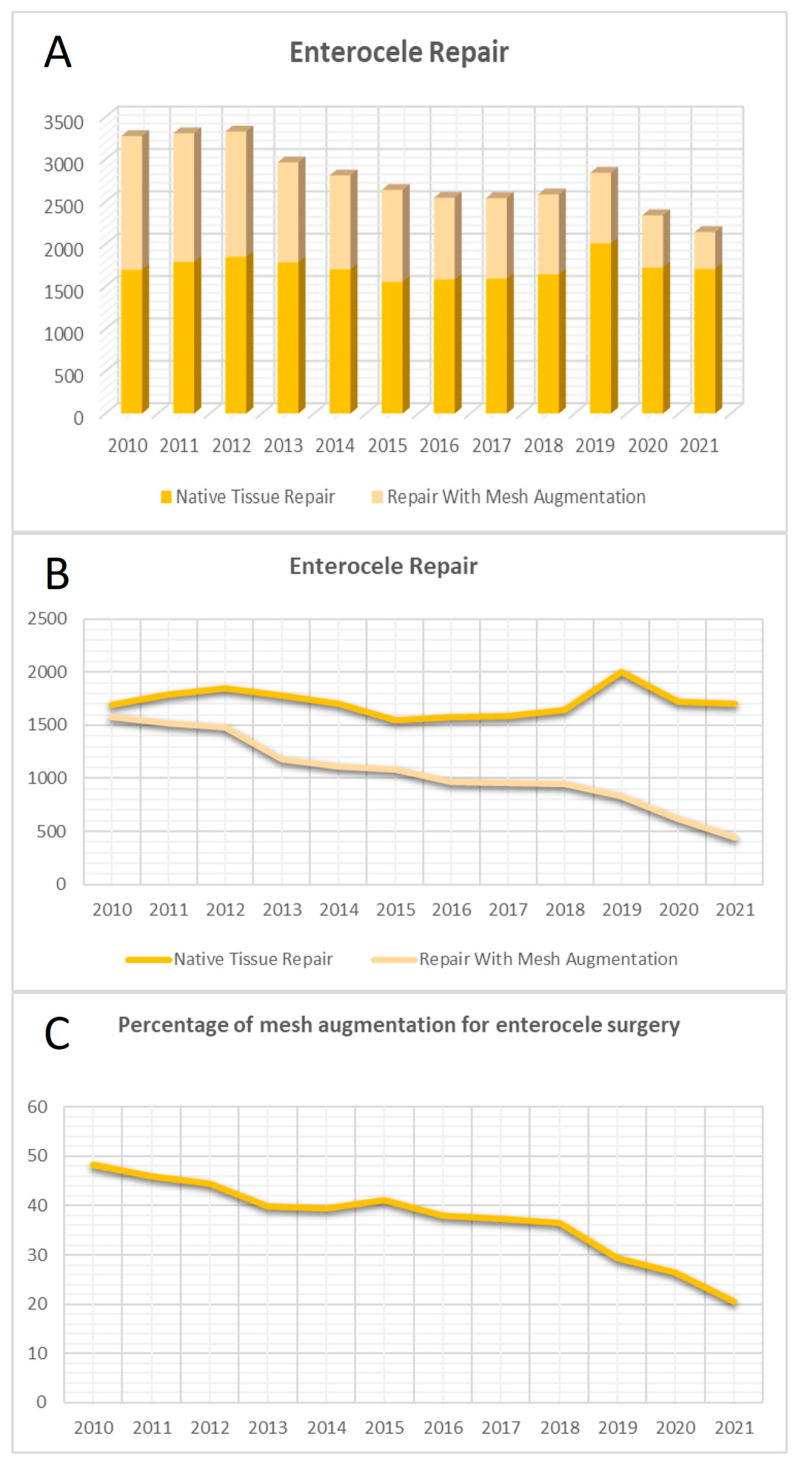
Surgical trends in enterocele repair. (**A**) Total number of enterocele repairs over the years. (**B**) Curves of the surgeries with native tissue repair (NTR) and transvaginal mesh (TVM) for enterocele repair. (**C**) Curve of the proportion of TVM surgeries for enterocele repair.

From 2010 onwards, enterocele correction via TVM and NTR received specific OPS codes. The trend in the corresponding curve for the total number of enterocele surgeries indicates a significant decrease from 2010 to 2021 (β = −0.896, *p* < 0.001, R^2^ = 0.80). This decline is attributed to the decreasing use of TVM for enterocele repair over the years. In comparison, the NTR curve remained relatively stable (NTR: β = −0.042, *p* = 0.898, R^2^ = 0.002), while the mesh curve exhibited a decreasing trend (TVM: β = −0.973, *p* < 0.001, R^2^ = 0.94). Associated with this, the proportion of TVM for enterocele repair experienced a significant decline over time (β = −0.943, *p* < 0.001, R^2^ = 0.89) (Figure 3).

**Figure 4:**
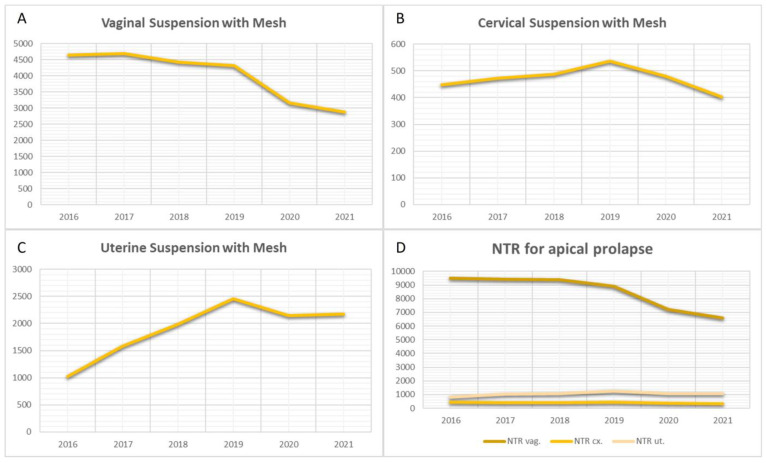
Surgical trends in apical pelvic organ prolapse. (**A**) Vaginal suspension with Mesh. (**B**) Cervical suspension with Mesh. (**C**) Uterine suspension with Mesh. (**D**) Native tissue repair (NTR).

Another application of TVM is in the repair of apical pelvic POP through the vaginal approach. Due to the absence of specific codes, data for this procedure were included starting from 2016. The curves depicting apical fixation illustrate a significant decline in the use of TVM for vaginal stump fixation (β = −0.914, *p* = 0.011, R^2^ = 0.83). However, there is stability in cervical fixation (β = −0.181, *p* = 0.731, R^2^ = 0.03) and a slight, statistically significant increase in uterine fixation, indicative of uterine-preserving POP surgery (β = 0.821, *p* = 0.045, R^2^ = 0.67) (Figure 4). Additionally, the curves for NTR data related to apical POP demonstrated relative stability in cervical and uterine fixation. However, a significant decline in vaginal stump fixation was observed from 2018 onwards (β = −0.908, *p* = 0.012, R^2^ = 0.82) (Figure 4).

**Figure 5:**
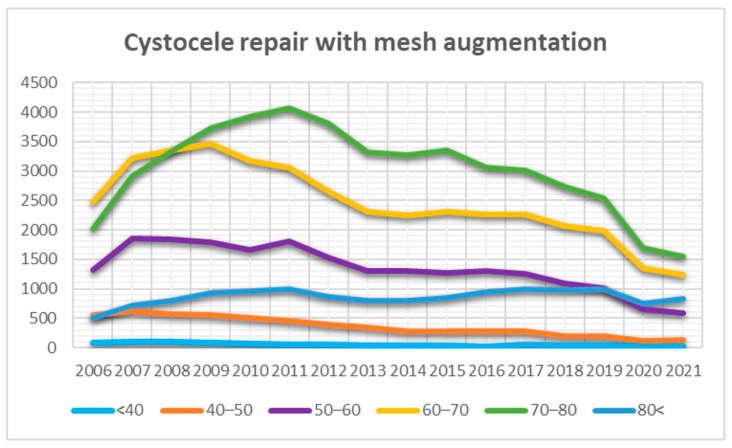
Trends in transvaginal mesh (TVM) surgeries within the anterior compartment observed across various age categories over the years.

Additional analyses conducted across various age groups for TVM surgery revealed a decreasing trend in TVM surgery in the anterior compartment over time among women under 50 years, becoming a rarity. The use of TVM surgery increased among older patients (80<) and showed a relatively fluctuating but generally decreasing trend among women aged between 50 and 80 (Figure 5).

**Figure 6:**
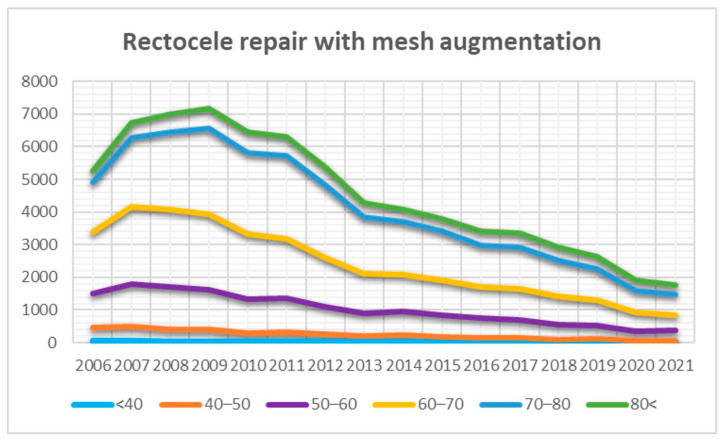
Trends in transvaginal mesh (TVM) surgeries within the posterior compartment observed across various age categories over the years.

**Figure 7:**
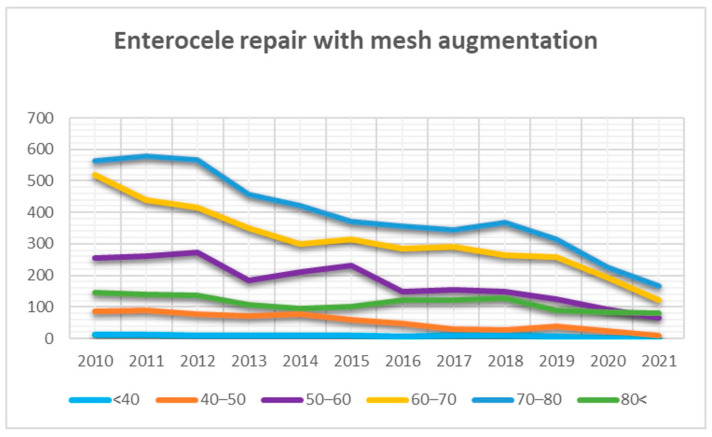
Trends in transvaginal mesh (TVM) surgeries for enterocele repair observed across various age categories over the years.

The curves representing TVM surgery in the posterior compartment showed a decreasing trend in each age group (Figure 6). Similarly, the results for enterocele correction showed a decreasing trend in most age groups, except for women aged over 80 years, where TVM surgery for enterocele correction remained relatively stable despite a small number (Figure 7).

**Figure 8:**
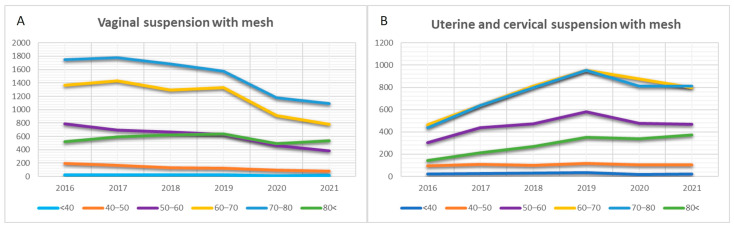
Trends in transvaginal mesh (TVM) surgeries for apical pelvic organ prolapse (POP) observed across various age categories over the years. (**A**) Vaginal suspension with mesh. (**B**). Uterine and cervical suspension with mesh.

The curves depicting apical fixation of the vaginal stump tended to decrease for each age group, with the trend remaining relatively stable for women aged over 80 (Figure 8). Cervical or uterine fixation using TVM as a uterine-preserving option showed an increasing trend in the respective curves for women over 50 years old. However, this option remained uncommon among women under 50 (Figure 8).

## 4. Discussion

Since the early 2000s, the utilization of mesh surgery exhibited a consistent global increase until the issuance of the initial FDA warning in 2008, triggered by a simultaneous surge in complications associated with transvaginal mesh procedures [15].

The FDA defines meshes as therapeutic devices intended for the reinforcement or repair of compromised or damaged tissues. These devices are methodically classified into three distinct categories based on their risk profile and regulatory requirements. Group I includes devices characterized by a low-risk profile, subject to general controls which include adherence to standard manufacturing practices and marketing regulations. Group II devices, presenting a marginally higher risk compared to Group I, necessitate additional specialized investigations. The most stringent category, Group III, includes high-risk devices, for which extensive preclinical evaluations are imperative prior to obtaining regulatory clearance [16].

Implantable devices such as transvaginal meshes (TVMs), originally classified under Class II, underwent the approval process through Section 510(k) of the Food, Drug, and Cosmetic Act. This procedural pathway requires manufacturers to submit a premarket notification to the FDA at least 90 days before the proposed marketing commencement. Central to Section 510(k) is the demonstration of “substantial equivalence” to existing devices (referred to as predicates) in the market. Notably, the first FDA approval for a product developed for pelvic organ prolapse (POP) repair was granted in 2002 [10]. This initial classification of TVMs under Group II allowed manufacturers to bypass rigorous approval studies, operating under the assumption of equivalence to previously approved mesh products for abdominal hernia treatment. This led to a scenario where several of these products were introduced into the market without pre-marketing studies evaluating safety and effectiveness. Remarkably, for many devices, no clinical trials were published at the time of their approval [17]. During this period, a considerable influx of such products was observed, with TVM rapidly gaining popularity in the United States as a prevalent surgical intervention for POP across various age demographics [15].

The initial surge in TVM use was met with growing concern, particularly after the FDA’s 2008 warning, triggered by an escalation in reported adverse events associated with TVM surgeries. The FDA undertook a comprehensive review of literature from 1996 to 2011, leading to the conclusion that transvaginal mesh exhibited a higher complication rate than initially anticipated. Following this, the FDA released a second safety communication, intensifying the spotlight on the risks associated with mesh implantations [12,18].

The impact of the FDA’s first warning in 2008 resonated within the clinical community, leading to a plateau in the use of transvaginal meshes in Anglo-Saxon countries, including England and the USA [14]. The subsequent FDA warning in 2011, however, resulted in a substantial reduction in transvaginal mesh usage, initiating a shift in numerous countries. In the USA, a study indicated that the utilization of transvaginal mesh for prolapse repairs decreased from 27% in early 2008 to 15% at the time of the first FDA warning, further dropping to 5% after the second FDA notification and reaching 2% by the end of 2011 [19]. The findings from another database study in the USA demonstrated a plateau post-2008 and a decline in vaginal mesh surgeries after 2011 [20].

The data collected during the development of TVM surgery yield critical insights into its utilization trends, particularly in the context of pelvic organ prolapse (POP) treatment. Analysis indicates a marked decline in the employment of TVM for POP post-2011, subsequent to the issuance of the FDA report [21]. This trend shift reflects a more judicious and discerning approach to TVM indication following the FDA warning, contrasting with a previously more liberal and indiscriminate application.

Further scrutiny, particularly of the Bloomberg Law Database spanning from 2000 to 2014 and relevant legal documentation, elucidates the professional credentials of clinicians involved in TVM implantation. Notably, within the framework of TVM-related litigation cases, a mere 12% of the implanting surgeons were verified or subsequently attained board certification in Female Pelvic Medicine and Reconstructive Surgery [22]. This statistic potentially implies a prior prevalence of inadequate expertise in TVM application, particularly before the dissemination of FDA advisories. Moreover, the immediate aftermath of the FDA reports witnessed a notable escalation in surgical interventions for the revision or removal of TVM and meshes utilized in stress urinary incontinence treatment. Intriguingly, this surge occurred despite the FDA warning explicitly targeting meshes used in prolapse treatment [23]. This phenomenon may be indicative of a broader, reflexive response within the medical community to heightened regulatory scrutiny and emerging safety concerns surrounding mesh applications.

A comprehensive public database study from England illustrated a persistent decrease in transvaginal mesh use following the initial FDA warning in 2008, dropping from 5.6% for anterior and posterior colporrhaphies to 1.5% in 2016 [14]. In 2016, in response to accumulating evidence and concerns regarding the safety and efficacy of TVM, the FDA revised the risk classification of these devices. This administrative action elevated TVM from a Class II to a Class III medical device, a category reserved for high-risk devices. The reclassification mandated the execution of rigorous clinical studies to substantiate safety and effectiveness prior to their market introduction. Despite this increased regulatory stringency, a notable development occurred in 2019. The FDA, citing the lack of comprehensive follow-up data, opted for a more drastic regulatory measure by imposing a prohibition on the use of transvaginal meshes in the United States [18]. This decision to ban the meshes was somewhat unexpected, considering the previously heightened oversight and the absence of new follow-up data explicitly indicated by the FDA. The reduction in transvaginal mesh utilization was succeeded also by a further market ban in England, Australia, and New Zealand [24]. Nevertheless, there are country-specific variations in the response to the FDA warning, exemplified by Portugal, where usage even slightly increased after the first warning [25]. Vaginal meshes continue to be used in selected patient groups in numerous European, Asian, and South American countries [18,26].

Our comprehensive database study, including a high amount of operations aligned with OPS codes, revealed a substantial decrease in the overall volume of TVM surgeries conducted in both the anterior and posterior compartments. In contrast, we showed a slight increase in TVM procedures in the apical compartment, suggesting a trend toward uterine-preserving interventions. However, the absence of specific codes limits a more extensive long-term analysis of mesh utilization in the apical compartment, allowing only for a limited examination conducted between 2016 and 2021. Previous studies have indicated that up to 60% of women expressed a preference for uterine preservation when seeking treatment for prolapse [27,28], and evidence suggested that hysterectomy did not result in superior success rates over uterine-preserving approaches [2]. The observed trend in apical prolapse correction with TVM seems to align with this evidence.

Notably, the proportion of mesh usage in the anterior compartment seems to have maintained a relatively stable trajectory over the years. The corresponding German–Austrian–Swiss guideline for POP treatment, which referred to the study period, provided data demonstrating that the recurrence rate in the anterior compartment subsequent to colporrhaphies without transvaginal mesh is threefold higher compared to transvaginal mesh repair [29].

Due to significant shifts in the landscape of marketed TVM products, in response to the FDA warnings, the German–Austrian–Swiss guidelines for POP underwent a series of revisions corresponding to this period of change. These updates included the incorporation of additional studies and the latest evidence available up to the year 2014, prior to the finalization and publication of the guidelines. It is critical to acknowledge that the systematic review conducted by the FDA, which played a pivotal role in shaping global perception and regulatory approaches towards TVM, was based on scientific literature available up to 2011. It is noteworthy that many of the TVM products scrutinized during this period have since been withdrawn from the market. Further developments in 2016 saw the Federal German Institute for Drugs and Medical Devices reference a publication by the Scientific Committee on Emerging and Newly Identified Health Risks (SCENIHR). This publication underscored the continued importance of TVM as a vital treatment modality for POP, albeit under specific indications. The German Institute, recognizing the significance of this finding, endorsed this perspective and provided a corresponding opinion with a link on its official website [30]. This development highlights the evolving nature of the clinical and regulatory landscape surrounding the use of TVM in POP treatment, influenced by ongoing research and safety evaluations. In contrast to findings from other studies, the prevalence of TVM procedures in the anterior compartment in Germany has demonstrated relative stability over the past two decades [14,19,25,31]. Meta-analyses suggested that transvaginal mesh surgery in the anterior compartment yielded higher anatomical cure and satisfaction rates. However, no significant differences were observed in re-operation rates, subjective cure, and postoperative quality of life [32,33]. Further analyses conducted in our study, stratified by different age groups, revealed a marginal increase in the utilization of TVM among women aged over 80. Additionally, we observed a subtle plateau following a significant decrease in women over 50. Conversely, transvaginal surgery in the anterior compartment for younger individuals remained a rare surgical procedure, exhibiting a modest decline.

Higher recurrence rates after surgical repair in the anterior compartment are associated with risk factors, such as major defects in pelvic floor support structures, levator avulsion, and abnormal distensibility of the levator hiatus, as well as a history of previous unsuccessful pelvic floor surgery and a large prolapse before surgery [9,34,35]. The adoption of TVM, following thorough counseling, for these specific populations characterized by a high risk of relapse and contraindications to alternative surgical approaches seems to remain an acceptable option.

Moreover, the trend in transvaginal mesh repair for the posterior compartment showed a consistent decline from 2008–2009. Despite transvaginal repair being considered superior to transanal repair for rectocele [3], the supplementary utilization of transvaginal mesh in the posterior compartment does not confer advantages over native tissue repair and is currently not recommended [36,37].

Our analysis demonstrated that the use of transvaginal mesh in the posterior compartment has experienced a significant decrease, particularly among women over 50, since 2008. Additionally, this approach remains a rarity in women under 50. The trends in enterocele correction similarly showed a decreasing use of transvaginal mesh, with the exception of women over 80. However, surgical interventions in this age group are notably uncommon. In our analytical evaluation, a discernible, albeit slight, increase was observed in the trend curves pertaining to individuals aged over 80 years. This increase can be primarily attributed to a higher likelihood of recurrence intervention correlated with advancing age. Within this specific demographic, it is plausible to postulate an increased prevalence of women who have undergone treatment for POP recurrence. Nevertheless, it is pertinent to acknowledge that our study encountered a limitation in the form of insufficient data pertaining to surgical indications. Consequently, while this hypothesis regarding a higher rate of recurrence interventions in the older age cohort remains tenable, we cannot provide a clear depiction of this trend in our analysis due to the lack of comprehensive data regarding surgical indications.

To elucidate the relationship between vaginal NTR and transvaginal mesh repair for apical prolapse, we conducted an analysis of corresponding curves for NTR. The data indicated a slight decrease for NTR from 2018 onwards. Despite the rarity of uterine fixation through vaginal native tissue repair, the trend for this procedure exhibited a marginal increase. Conversely, the fixation of the vaginal stump with mesh displayed a declining trend across all age groups.

The decline observed in both NTR and TVM repair may be attributed to the expanding availability of alternative treatment modalities for apical prolapse. Sacrocolpopexy has emerged as the gold standard for surgical intervention in vaginal vault prolapse [38]. Evidence suggests that sacrocolpopexy achieves comparable anatomical and subjective success rates to transvaginal mesh surgery, with potential advantages in terms of mesh-related complication rates [39]. Consequently, the rates of apical prolapse repair through sacrocolpopexy have steadily increased over the last two decades [40,41]. However, despite this trend, the continuous evolution of corresponding products and the option of uterine-preserving surgery with transvaginal mesh, among other vaginal approaches showing over 85% success rates [13], positions the TVM procedure as an option for uterine preservation among women over 50 years.

The impact of the FDA warnings on the global landscape of TVM surgery has been diverse and multifaceted. In 2014, a significant regulatory shift occurred in the UK and Scotland, where a ban on TVM was implemented pending the availability of robust evidence [42]. The global discourse on TVM, heightened in 2009, led to the initiation of the multicenter UK trial “PROSPECT.” The findings of this trial significantly influenced national guidelines, particularly those issued by the National Institute for Health and Care Excellence (NICE) in 2017, which recommended limiting TVM repairs to clinical study [18]. However, the PROSPECT study faced critique regarding its methodology, notably for permitting the use of both lightweight and heavyweight meshes and the variable surgical expertise of the participating surgeons. The trends in TVM surgery in other Anglo-Saxon countries have also evolved over time, influenced by the UK’s experiences and FDA warnings [18]. In contrast, a long-term study from Asia presents a different trajectory, indicating an increase in TVM surgery for POP from 2004 to 2018. The authors of this study attributed this rise to enhanced surgical proficiency and reduced complications, attributed to the employment of lighter mesh products by specialist surgeons, reflecting current evidence-based practices [43]. Further data from Asia suggest improved outcomes in TVM surgeries when conducted by trained professionals using lighter mesh products [44,45]. Conversely, a comprehensive study from Latin America (LATAM) reveals a different scenario. Despite a lack of precise data on TVM surgery trends, the study indicates that nearly 80% of POP specialists in LATAM continue to advocate for mesh-based repairs in specific cases, suggesting the persistent use of TVM in these regions despite the international developments following FDA warnings [26]. The study authors hypothesize that the lower incidence of legal disputes related to mesh complications in LATAM, as compared to the US, may partly account for the continued widespread adoption of mesh repairs in these countries.

Over the previous decade, the landscape of TVM usage in Germany has undergone a considerable transformation. Notably, the meshes mentioned in the FDA report have been excluded from the German market. These have predominantly been replaced by lightweight meshes, which, as per recent scholarly publications, demonstrate significantly reduced rates of exposure [46]. A pivotal development in this context is the initiation of a certification program for physicians by the German Association of Urogynecologists (AGUB). This program is centered around enhancing proficiency in urogynecology. Furthermore, prominent societies such as the German Association of Obstetrics and Gynecology (DGGG) and the German Continence Society have been vocal advocates for the establishment of specialized centers dedicated to this field. The implementation of these centers ensures the adherence to guidelines in the utilization of TVM implants, thereby fostering a standardized approach [18].

The evolving pattern of TVM surgery in Germany can arguably be attributed to such systemic standardization. This has led to a more structured and guideline-conformant use of transvaginal meshes, ensuring that indications for their use are in line with established medical guidelines and best practices.

Noteworthy, we observed a slight decreasing trend across all assessed surgical procedures, starting from the year 2019. In late 2019, the coronavirus disease in 2019 (COVID-19) was first reported in China, rapidly spreading to other nations. By early 2020, COVID-19 had met the criteria for a pandemic, prompting the World Health Organization to declare it a “public health emergency of international concern” on 30 January 2020 [47]. In adherence to recommendations from various urogynecological societies, the volume of urogynecological surgical procedures in German clinics experienced a significant reduction during the subsequent pandemic and lockdown.

However, it is important to acknowledge several limitations of this study. While the German Federal Statistical Office provides OPS codes and corresponding numerical data for in-patient procedures, the accuracy of these data cannot be validated in the absence of case-specific information. The absence of operation indications, along with data concerning concomitant surgeries and primary and recurrent procedures, imposes constraints on the depth of this analysis. Despite these limitations, the major strength of our study lies in the comprehensive nationwide data available, presented over multiple years, enabling the demonstration of a long-term trend.

## 5. Conclusions

Transvaginal mesh surgery for POP has been a subject of extensive debate, characterized by increasing complication rates and multiple warnings from the FDA. Despite evolving global trends, transvaginal mesh implants persist as an option for POP surgery. The decision to opt for TVM surgery should be individualized, guided by experienced surgeons, in accordance with recommendations from the SCENIHR and the German–Austrian–Swiss guideline, particularly for patients with specific clinical characteristics.

Our study results, elucidating the shifts in trends in transvaginal mesh surgery, are anticipated to empower clinicians to make informed decisions tailored to the unique needs of each patient, thereby enhancing the overall quality of care. Furthermore, we advocate for the development of training programs that align with these evolving trends. Additional research efforts are warranted to comprehensively understand the ongoing trend and identify its underlying determinants.

## Figures and Tables

**Table 1:** OPS codes for transvaginal pelvic organ prolapse (POP) surgeries and their corresponding procedures.

OPS-Codes	Procedures
5-704.00	Anterior colporrhaphy without alloplastic material
5-704.01	Anterior colporrhaphy with alloplastic material
5-704.10	Posterior colporrhaphy without alloplastic material
5-704.11	Posterior colporrhaphy with alloplastic material
5-704.4g	Vaginal cuff fixation (vaginal) with alloplastic material
5-704.4e-f	Vaginal cuff fixation (vaginal) without alloplastic material (Ligg. Sacrouterinae, Lig. sacrospinalis or Lig. Sacrotuberale)
5-704.5g	Cervical stump fixation (vaginal) with alloplastic material
5-704.5e-f	Cervical stump fixation (vaginal) without alloplastic material (Ligg. sacrouterinae, Lig. sacrospinalis or Lig. sacrotuberale)
5-704.6a	Uterus fixation (vaginal) with alloplastic material
5-704.68-69	Uterus fixation (vaginal) without alloplastic material (Ligg. Sacrouterinae, Lig. sacrospinalis or Lig. Sacrotuberale)
5-707.21	Enterocele repair (vaginal) without alloplastic material
5-707.31	Enterocele repair (vaginal) with alloplastic material

## Data Availability

All data are included in the study.
